# Parkin loss of function contributes to RTP801 elevation and neurodegeneration in Parkinson's disease

**DOI:** 10.1038/cddis.2014.333

**Published:** 2014-08-07

**Authors:** J Romaní-Aumedes, M Canal, N Martín-Flores, X Sun, V Pérez-Fernández, S Wewering, R Fernández-Santiago, M Ezquerra, C Pont-Sunyer, A Lafuente, J Alberch, H Luebbert, E Tolosa, O A Levy, L A Greene, C Malagelada

**Affiliations:** 1 Department of Pathological Anatomy, Pharmacology and Microbiology, Faculty of Medicine, Universitat de Barcelona, Barcelona, Catalonia, Spain; 2 Department of Pathology and Cell Biology, Columbia University, New York, NY, USA; 3 Department of Animal Physiology, Faculty for Biology, Ruhr University, Bochum, Germany; 4 Laboratory of Neurodegenerative Disorders and Department of Neurology, Institut Clínic de Neurociències, Hospital Clínic de Barcelona, Department of Medicine, Universitat de Barcelona, Barcelona, Catalonia, Spain; 5 IDIBAPS-Institut d'Investigacions Biomèdiques August Pi i Sunyer, Centro de Investigación Biomédica en Red sobre Enfermedades Neurodegenerativas (CIBERNED), Barcelona, Catalonia, Spain; 6 Department of Cell Biology, Immunology and Neurosciences, Faculty of Medicine, Universitat de Barcelona, Barcelona, Catalonia, Spain; 7 Department of Neurology Columbia University, New York, NY, USA

## Abstract

Mutations in the *PARK2* gene are associated with an autosomal recessive form of juvenile parkinsonism (AR-JP). These mutations affect parkin solubility and impair its E3 ligase activity, leading to a toxic accumulation of proteins within susceptible neurons that results in a slow but progressive neuronal degeneration and cell death. Here, we report that RTP801/REDD1, a pro-apoptotic negative regulator of survival kinases mTOR and Akt, is one of such parkin substrates. We observed that parkin knockdown elevated RTP801 in sympathetic neurons and neuronal PC12 cells, whereas ectopic parkin enhanced RTP801 poly-ubiquitination and proteasomal degradation. In parkin knockout mouse brains and in human fibroblasts from AR-JP patients with parkin mutations, RTP801 levels were elevated. Moreover, in human postmortem PD brains with mutated parkin, nigral neurons were highly positive for RTP801. Further consistent with the idea that RTP801 is a substrate for parkin, the two endogenous proteins interacted in reciprocal co-immunoprecipitates of cell lysates. A potential physiological role for parkin-mediated RTP801 degradation is indicated by observations that parkin protects neuronal cells from death caused by RTP801 overexpression by mediating its degradation, whereas parkin knockdown exacerbates such death. Similarly, parkin knockdown enhanced RTP801 induction in neuronal cells exposed to the Parkinson's disease mimetic 6-hydroxydopamine and increased sensitivity to this toxin. This response to parkin loss of function appeared to be mediated by RTP801 as it was abolished by RTP801 knockdown. Taken together these results indicate that RTP801 is a novel parkin substrate that may contribute to neurodegeneration caused by loss of parkin expression or activity.

Parkinson's disease (PD) is among the most frequent neurodegenerative disorders, characterized by loss of specific populations of neurons in both the central and peripheral nervous systems, including those in the substantia nigra pars compacta (SNpc) and sympathetic ganglia.^1, 2, 3^ Although treatments to ameliorate clinical manifestations of PD are common, there are no pharmacological therapies to suppress neuron degeneration and death.^4^

The *PARK2* gene encodes for parkin protein. Parkin is an E3 ligase and genetic mutations impair its enzymatic activity and solubility. These *PARK2* mutations are linked to the appearance of an autosomal recessive form of juvenile parkinsonism (AR-JP).^5, 6^

Apart from mutations, parkin E3 ligase activity can be inactivated both *in vitro* and *in vivo* by S-nitrosylation,^7^ oxidative stress^8^ and dopaminergic stress.^9^ The combination of these stresses plus heterozygous parkin mutations can also lead to earlier manifestations of parkinsonism.^10^ AR-JP symptomatology resembles sporadic PD, with loss of neuromelanin positive (NM+) catecholaminergic neurons in the SNpc and locus coeruleus.

Parkin overexpression or restoration of parkin activity in culture or in animal models protects from various neurodegenerative conditions including mutant alpha synuclein,^11^ kainic acid^12^ and 6-hydroxydopamine (6-OHDA) toxicity.^13, 14^ In Drosophila, parkin has been linked to protein translation by interacting with the TSC/TOR/4EBP pathway.^15^ One upstream regulator of the TSC/TOR/4EBP pathway is *DDIT4*, a stress-regulated gene that encodes a protein designated RTP801/REDD1 that negatively regulates the mechanistic target of rapamycin (mTOR).^16, 17^ In cellular models of PD, *DDIT4* was the most highly upregulated transcript (98-fold) and its encoded protein, RTP801, was significantly induced.^18^ Moreover, RTP801 was upregulated in animal models of PD and was elevated in NM+ neurons in the SNpc of idiopathic PD patients in comparison with non-PD controls.^19^

RTP801 is both sufficient and necessary to mediate neuron death in *in vitro* and *in vivo* models of PD.^19^ This involves a sequential mechanism in which it first blocks mTOR activation and then, as a consequence, leads to the inactivation of the neuronal survival kinase Akt, which is also an mTOR substrate.^20^

As RTP801 protein has a very short cellular half-life (2–5 min)^21, 22, 23^ and is subject to fine-tuned regulation, we investigated whether parkin contributes to RTP801 degradation. We first explored whether RTP801 is a parkin substrate, and second, whether parkin loss of function leads to a toxic accumulation of RTP801 that could contribute to neurodegeneration.

## Results

### RTP801 is degraded by the proteasome and poly-ubiquitinated by parkin

**Figure 1:**
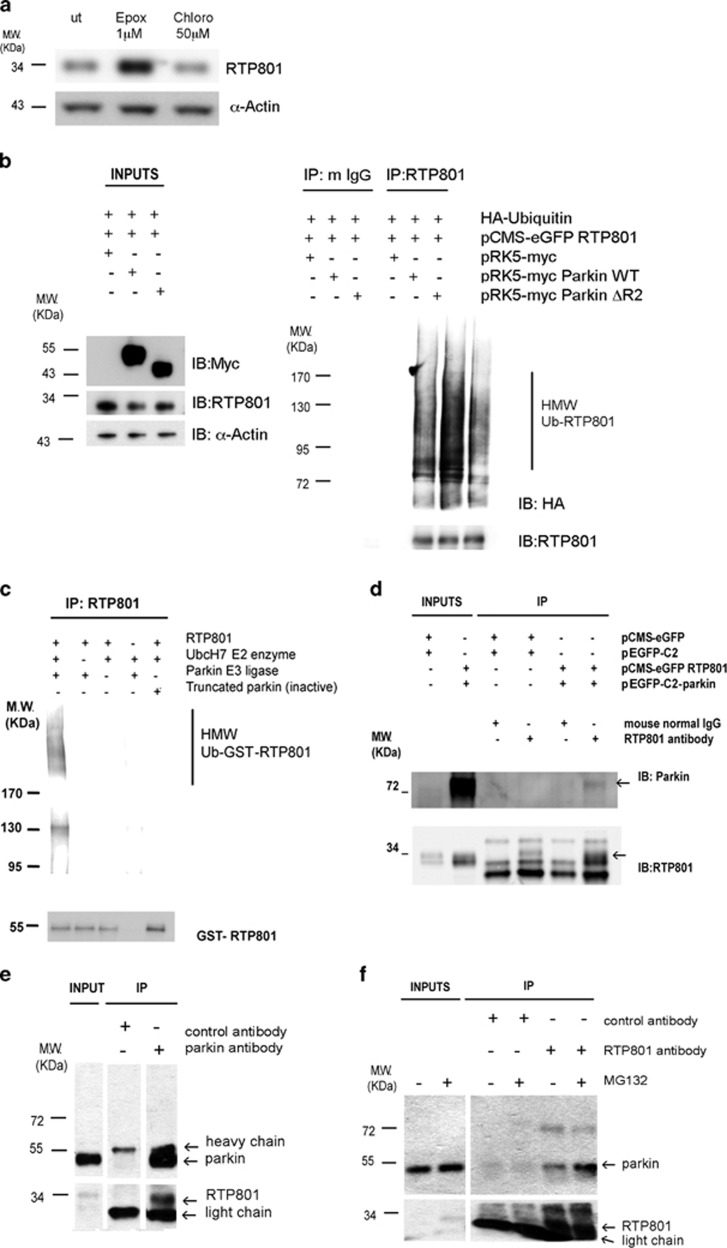
RTP801 is poly-ubiquitinated by parkin E3 ligase and degraded by the proteasome. (**a**) RTP801 is degraded by the proteasome. HEK293 cells were exposed for 4 h to 1 *μ*M epoxomycin or 50 *μ*M chloroquine, and cell lysates were analyzed by western immunoblotting for RTP801 and *α*-actin (loading control). ut = untreated. (**b**) RTP801 is poly-ubiquitinated by parkin prior to proteasomal degradation. HEK293 cells were co-transfected with pCMS-eGFP RTP801, HA-ubiquitin and one of the pRK5-myc constructs, either empty or containing parkin WT or parkin ΔR2. Twenty-four hours later, cultures were exposed to epoxomycin for 3 h prior to harvesting. RTP801 was immunoprecipitated (IP) and immunocomplexes along with whole-cell lysates were analyzed by western immunoblotting (IB). Membranes were probed for HA, myc and *α*-actin as a loading control. Membranes were reprobed with anti-RTP801 antibody to confirm that RTP801 was immunoprecipitated. Non-specific mouse immunoglobulins were used as negative controls (m IgG). (HMW Ub-RTP801, high molecular weight ubiquitinated RTP801) (**c**) Parkin ubiquitinates RTP801 in a cell-free *in vitro* system. Recombinant parkin E3 ligase (active full-length), truncated parkin (inactive), GST-RTP801, and UbcH7 E2 enzyme, were mixed and incubated as indicated along with biotinylated ubiquitin, E1 enzyme, and ATP. RTP801 was immunoprecipitated, and immunocomplexes resolved in a western blot. The membrane was incubated with Avidin/Biotin and then incubated with chemiluminiscent peroxidase substrate solution (upper panel). The same membrane was reprobed with an anti-RTP801 antibody (lower panel). (HMW Ub-RTP801, high molecular weight ubiquitinated RTP801) (**d**) Ectopic parkin physically interacts with ectopic RTP801. HEK293 cells were co-transfected either with the pCMS-eGFP and pEGFP-C2 empty vectors or pCMS-eGFP RTP801 along with pEGFP-C2-parkin. Twenty-four hours later, cultures were treated with the cross-linking agent DSP for 2 h at 4°C, prior to harvesting. After RTP801 immunoprecipitation, the samples were analyzed by western immunoblotting for parkin to detect the interaction, and for RTP801 as an IP control. (**e** and **f**) Reciprocal co-immunoprecipitation indicates interaction of endogenous parkin and RTP801 in cells. Neuronal PC12 cells were treated with or without MG132 for 5 h as indicated, lysed and subjected to immunoprecipitation (IP) with control antibody (GFP), and either (**e**) anti-parkin or (**f**) anti-RTP801 antibodies. Immunoprecipitates were analyzed by western immunoblotting with anti-parkin and anti-RTP801 as indicated. In (**e**) all samples were analyzed on the same blot, but irrelevant intervening lanes were removed. In the right panel (**f**), the membrane was probed with a light chain-specific secondary antibody to diminish overlap of signal with co-immunoprecipitated RTP801. In each panel (**a**, **b**, **c**, **d**, **e** and **f**), a representative western blot is shown from a pool of at least two independent experiments. Specific bands are pointed out by arrows

Previous studies^21, 22, 23^ indicated that RTP801 protein has a brief half-life, between 2 and 5 min. Therefore, we first investigated how RTP801 is degraded using HEK293 cells. Cultures were treated with epoxomycin, a specific proteasome inhibitor, and chloroquine, an inhibitor of intralysosomal catabolism. Western immunoblotting (WB) indicated that RTP801 was degraded mostly by the proteasome (Figure 1a). In nerve growth factor (NGF)-differentiated PC12 cells, which model catecholaminergic neurons,^24^ we observed similar results with epoxomycin. Chloroquine exposure for 6 h, but not 30 h, mildly increased RTP801 levels (Supplementary Figure S1). These data confirmed that RTP801 degradation is mostly proteasomal in both HEK293 cells and NGF-differentiated PC12 cells.

Poly-ubiquitination involves three different enzymes, ubiquitin activator E1, ubiquitin transferor E2 and ubiquitin ligase E3. It also requires the presence of lysines in the target protein. RTP801 has six lysines that can be ubiquitinated. We assessed whether RTP801 is poly-ubiquitinated in cells by transfecting HEK293 cells either HA-tagged ubiquitin or with both HA-ubiquitin and pCMS-eGFP RTP801. Twenty-four hours after transfection, cultures were treated with epoxomycin for at least 2 h, and then both endogenous and ectopic RTP801 were immunoprecipitated. By WB, we detected high molecular weight RTP801 species with different lengths of poly-ubiquitin chains (HMW Ub-RTP801), for both endogenous (visible in the more exposed panel in Supplementary Figure S2) and ectopic RTP801. Therefore, our data indicate that RTP801 is poly-ubiquitinated and targeted to proteasomal degradation in living cells (Supplementary Figure S2).

Such results raised the question of which E3 ligases poly-ubiquitinate RTP801. One ligase particularly relevant to PD is parkin. Therefore, we investigated whether RTP801 poly-ubiquitination is enhanced by parkin, by transfecting HEK293 cells with RTP801 along with HA-ubiquitin and WT parkin or its inactive form parkin ΔR2, lacking the RING2 domain. By performing immunoprecipitation and WB, we detected high molecular weight RTP801 species with poly-ubiquitin chains (HMW Ub-RTP801) attached, and found that WT parkin but not its ΔR2-truncated form, substantially increased RTP801 poly-ubiquitination (Figure 1b). Therefore, our data indicate that RTP801 is poly-ubiquitinated in the presence of parkin and targeted to proteasomal degradation in living cells.

To address whether parkin directly poly-ubiquitinates RTP801, we used a cell-free assay. To reproduce the sequential poly-ubiquitination reaction, we combined recombinant parkin as the E3 ligase, recombinant GST-RTP801 as putative substrate, biotinylated ubiquitin, an E1 ubiquitin-activating enzyme, and E2 ubiquitin-conjugating enzyme UbcH7.^6, 25^ As negative controls, we performed the same reaction in the absence of either parkin, UbcH7, or GST-RTP801, or in the presence of inactive truncated parkin. WB analyses showed the appearance of high molecular weight species corresponding to poly-ubiquitinated GST-RTP801 (HMW Ub-GST-RTP801) only when parkin and all other constituents were present (Figure 1c, left lane).

### RTP801 and parkin interact in cells

Next, we assessed whether parkin and RTP801 interact in living cells. We overexpressed RTP801 and GFP-tagged parkin in HEK293 cells. After dithiobis succinimidyl propionate (DSP) cross-linking and immunoprecipitation with normal IgG or anti-RTP801, followed by WB, we observed co-immunoprecipitation of parkin with RTP801 (Figure 1d). We confirmed this interaction in the converse direction by immunoprecipitating ectopic parkin and probing for RTP801 (Supplementary Figure S3). Moreover, in neuronal PC12 cells, co-immunoprecipitation in both directions indicated that endogenous parkin interacts with endogenous RTP801 (Figures 1e and f). This interaction was observed when RTP801 was immunoprecipitated with or without pretreatment with the proteasome inhibitor MG132 (Figure 1f). Taken together, our results indicate that RTP801 interacts with parkin and that parkin promotes RTP801 poly-ubiquitination.

### Parkin regulates RTP801 levels in neuronal PC12 cells

**Figure 2:**
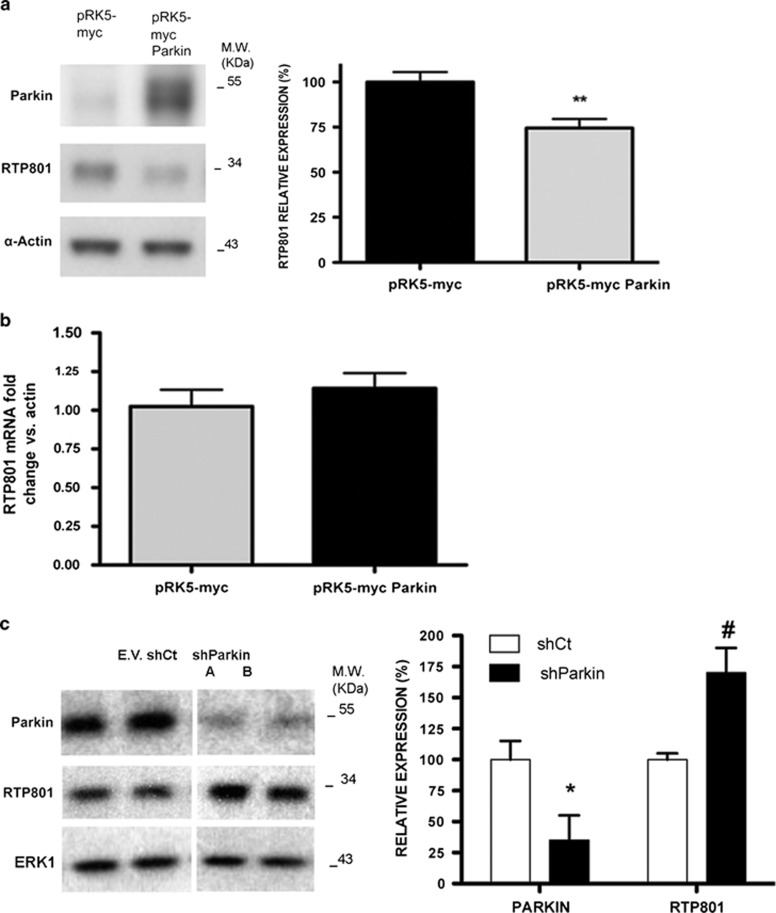
Parkin regulates RTP801 protein in neuronal PC12 cells. (**a**) Ectopic parkin decreases RTP801 protein levels. Neuronal PC12 cells were transfected with pRK5-myc or pRK5-myc Parkin constructs. Twenty-four hours later, cell extracts were harvested and subjected to western immunoblotting. Membranes were incubated with anti-parkin and anti-RTP801 antibodies and then reprobed with anti-*α*-actin antibody, as protein loading control. Right panel shows densitometries representing values as mean ± S.E.M. of at least three independent experiments. Student's *t*-test, **P<0.01 *versus* pRK5-myc. (**b**) Parkin does not regulate RTP801 transcriptionally. Neuronal PC12 cells were transfected with pRK5-myc or pRK5-myc Parkin constructs. RNA was extracted 20 h later, and reverse transcription-qPCR was performed to quantify RTP801 mRNA under the indicated conditions. Values represent mean ± S.E.M. of at least three independent experiments. (**c**) Parkin knockdown results in elevated expression of RTP801 protein. Neuronal PC12 cells were infected with lentiviruses containing an empty vector (EV), a scrambled negative control shRNA, or four different parkin shRNAs (results for shParkin A and B are shown on the left; knockdown by shParkin 1 ^33^ and 2 was comparable to that with shA and shB). Cell lysates were assessed 4 days later for RTP801 and parkin expression by western immunoblotting. Expression in each case was determined relative to the ERK1 loading control. Graph shows means of pooled data for all parkin shRNAs ± S.E.M. Student's *t*-test, *P<0.05 and ^#^*P*<0.05 *versus* shCt

As parkin poly-ubiquitinates RTP801 both *in vitro* and in cultured cells, we next asked whether parkin could directly regulate RTP801 levels in a cellular model. Consistent with this, transfected parkin significantly diminished basal levels of RTP801 in neuronal PC12 cells (Figure 2a). Note that the overall decrease in RTP801 was limited as transfection efficiency of neuronal PC12 cells was only 15–20%.

Several studies indicate that parkin has DNA-binding and transcriptional activities.^26, 27^ However, ectopic parkin did not affect RTP801 mRNA levels in neuronal PC12 cells, discounting the possibility that parkin regulates RTP801 at a transcriptional level (Figure 2b).

To further assess the role of parkin in RTP801 expression, we next diminished endogenous parkin expression with specific shRNAs delivered by lentiviral infection. In neuronal PC12 cells, shParkin elevated levels of RTP801 in comparison with empty vector or scrambled control shRNA by about 65–70% (Figure 2c).

We also confirmed the effect of shParkin sequences A and B on RTP801 expression by transient transfection in naive PC12 cells by WB (Supplementary Figure S4a). Additionally, NGF-differentiated PC12 cells transfected with these shRNAs were analyzed by immunofluorescence (Supplementary Figure S4b and c). Parkin knockdown (30–40%) significantly elevated RTP801 immunostaining (25–35%), although not as markedly as with lentiviral infection, owing to the lower efficiency of transfection (Supplementary Figure S4).

Taken together, these results indicate that parkin regulates endogenous RTP801 protein levels in neural cells and that parkin knockdown leads to an increase in RTP801 protein expression.

### Parkin deficiency *in vivo* elevates RTP801

**Figure 3:**
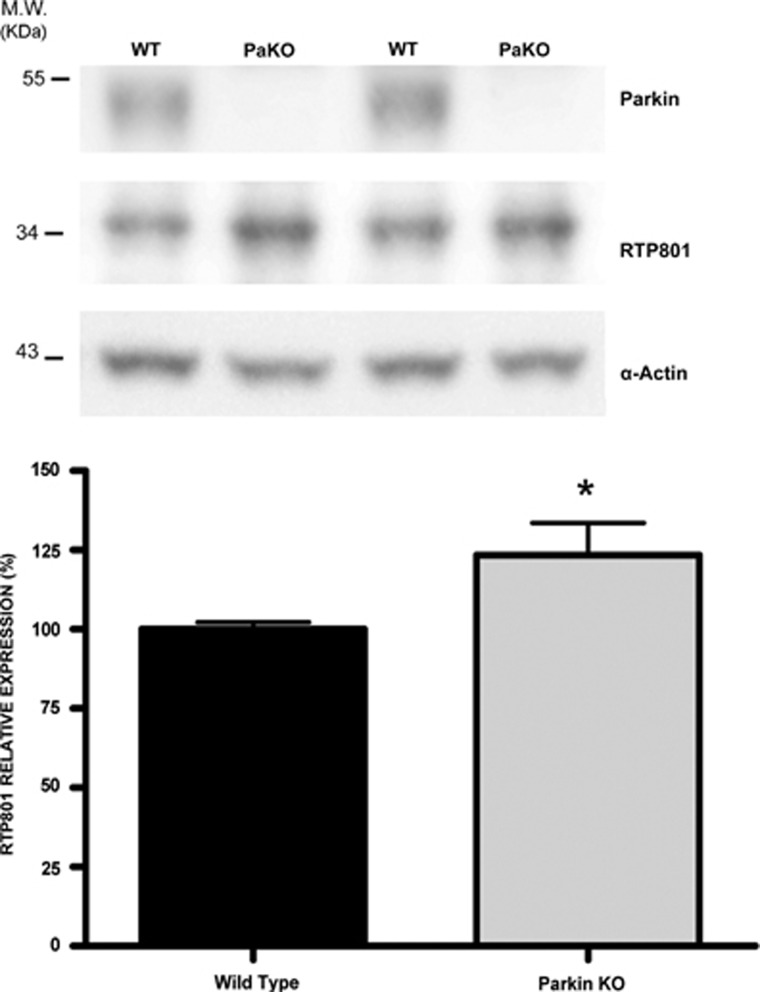
RTP801 is elevated in parkin knockout mouse brains. Whole-brain lysates from five 8-month-old PaKO mice and five wild-type littermates (WT) were analyzed by western immunoblotting. Membranes were incubated with anti-RTP801 and anti-parkin antibodies, and subsequently reprobed with anti-*α*-actin antibody as a loading control. Representative immunoblots are shown along with the densitometries representing values as mean ± S.E.M. of at least three independent membranes including all the samples. Student's *t*-test with Welch's correction, *P<0.05 *versus* WT

We next tested how loss of parkin affects RTP801 protein levels in a knockout (PaKO) mouse model with an exon 3 deletion in the *PARK2* gene.^28^ Although PaKO mice do not show a conventional parkinsonian phenotype, they are a useful model to study the role of parkin in protein regulation or oxidative stress.^29, 30^ By WB, we observed a modest but significant accumulation of RTP801 in 8-month-old PaKO mouse brains in comparison with wild-type littermates, confirming *in vivo* that parkin loss results in elevated RTP801 protein (Figure 3).

### RTP801 accumulates in AR-JP patient fibroblasts and in human postmortem PD brains with parkin mutations

**Figure 4:**
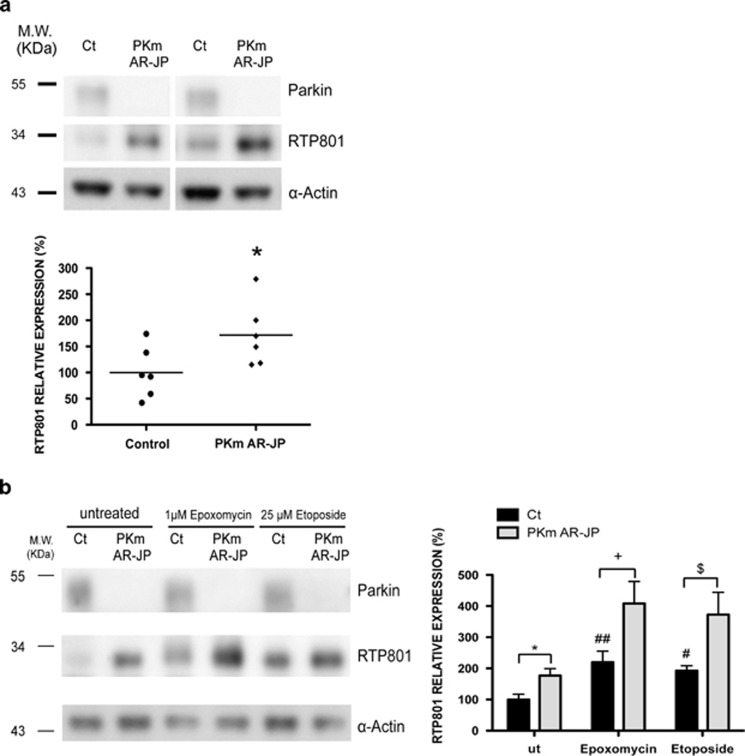
RTP801 is elevated in human fibroblasts derived from AR-JP patients with parkin mutations. (**a**) RTP801 is elevated in fibroblasts from parkin mutant AR-JP patients. Extracts from human primary fibroblasts derived from six diagnosed parkin mutant AR-JP patients (PKm AR-JP) and six control individuals (Ct) were subjected to western immunoblotting. Membranes were probed with antibodies against parkin and RTP801 and reprobed for *α*-actin (loading control). Representative immunoblots are shown along with the densitometries for RTP801 signals from at least three independent membranes including all the samples. Student's *t*-test, *P<0.05 *versus* control individuals. (**b**) Parkin mutant fibroblasts exposed to epoxomycin or etoposide show elevated RTP801 accumulation. The same human primary fibroblasts were treated for 4 h with 1 *μ*M epoxomycin or 25 *μ*M etoposide before being harvested. The resulting extracts were analyzed by western immunoblotting. Membranes were incubated with antibodies against parkin and RTP801, and subsequently reprobed with anti-*α*-actin antibody as a loading control. The graph shows densitometries for RTP801 signals from at least three independent membranes. Student's *t*-test, *P<0.05 *versus* control untreated, ^+^*P*<0.05 *versus* control epoxomycin, ^$^*P*<0.05 *versus* control etoposide; ANOVA with Bonferroni's multiple comparison test, ^#^*P*<0.05 and ^##^*P*<0.01 *versus* control untreated. ut = untreated

We next collected skin punches from AR-JP patients bearing exon deletions in the *PARK2* gene and from age-matched controls (see Supplementary Table 1), and used these to establish pure skin fibroblast cultures. By WB, we observed that parkin mutant fibroblasts possessed a significantly higher mean of RTP801 protein expression compared with controls (Figure 4a).

We next tested RTP801 protein expression in control and AR-JP fibroblasts after proteasome inhibition (epoxomycin) or DNA damage (etoposide). Etoposide is reported to cause a rapid and stable increase in RTP801 expression.^31^ After 4 h with these stressors, which had no effect on viability (data not shown), the RTP801 induction response of AR-JP cells was approximately double that in cells from control patients (Figure 4b). In conclusion, loss of parkin activity in mutant fibroblasts significantly elevates RTP801 protein both under basal and stressor conditions.

**Figure 5:**
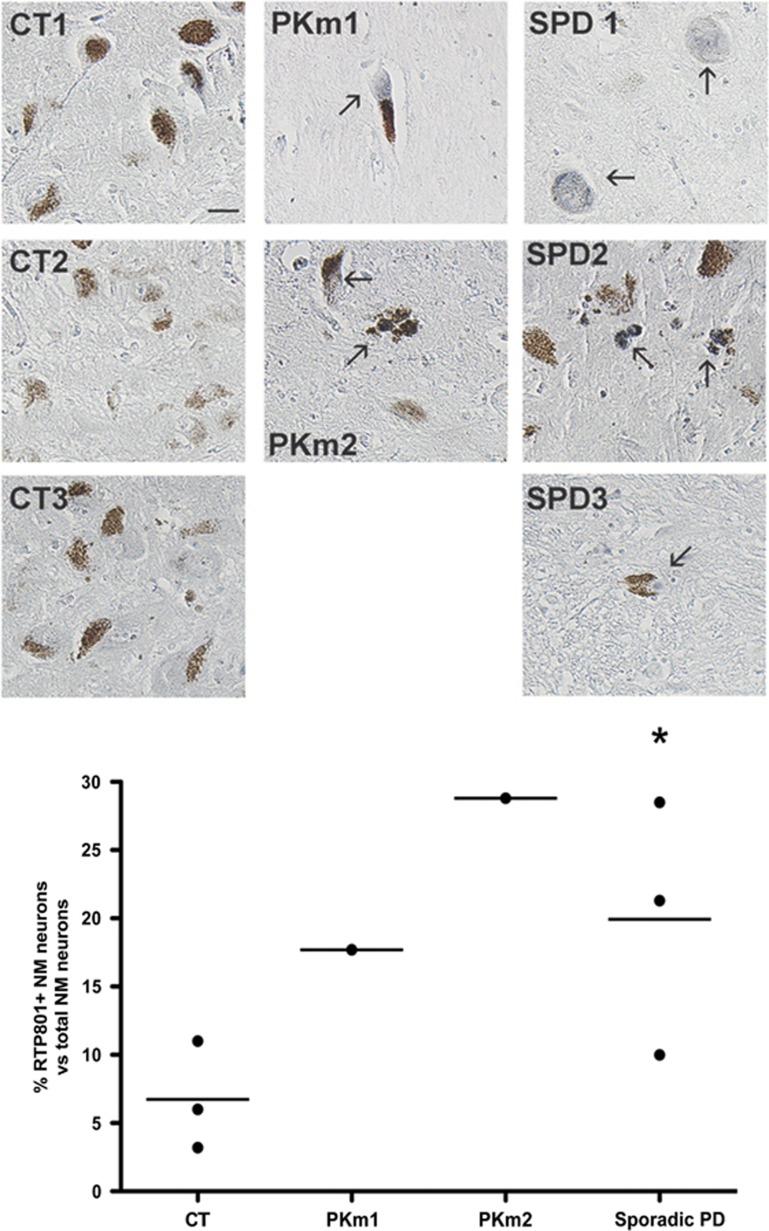
RTP801 is elevated in parkin mutant NM+ nigral neurons. Paraffin-embedded sections of SNpc from control individuals (CT1, CT2 and CT3), parkin simple heterozygous mutant patient (PKm1), parkin compound heterozygous mutant (PKm2) and sporadic PD patients (SPD1, SPD2 and SPD3) were immunostained for RTP801 (grey-blue). Note the presence of neuromelanin granules (brown) in the somas of dopaminergic neurons. Arrows point to highly stained NM+ nigral neurons. The percentage of NM+ and RTP801+ neurons *versus* total NM+ neurons was scored for each case, and results are represented in the graph. Student's *t*-test, *P<0.05 *versus* control individuals. In total, 150–500 neurons were evaluated per section

Our previous work demonstrated that RTP801 is upregulated in NM+ neurons in the SNpc of sporadic PD patients.^19^ Therefore, we next examined RTP801 expression in SNpc sections from patients bearing *PARK2* mutations. Only two cases with parkin mutations (one simple heterozygote, PKm1, and one compound heterozygote, PKm2) were available from the neurological tissue bank. We also included three cases of sporadic PD and three non-PD cases (see Supplementary Table 2). SNpc sections were immunostained for RTP801, and proportions of NM+ neurons highly stained for RTP801 were blindly scored. As previously observed, there was a very low proportion of RTP801+ nigral neurons in non-PD controls and this was significantly increased in sporadic PD cases and in the two cases with parkin mutations (Figures 5a and b). In contrast, little or no RTP801 staining was observed in cerebellum (a region little affected in PD^32^) from the same patients (Supplementary Figure S5). Taken together, these results indicate that parkin loss of function in humans correlates with elevated RTP801, in both fibroblasts and nigral neurons.

### Parkin protects neuronal PC12 cells from exogenous RTP801

**Figure 6:**
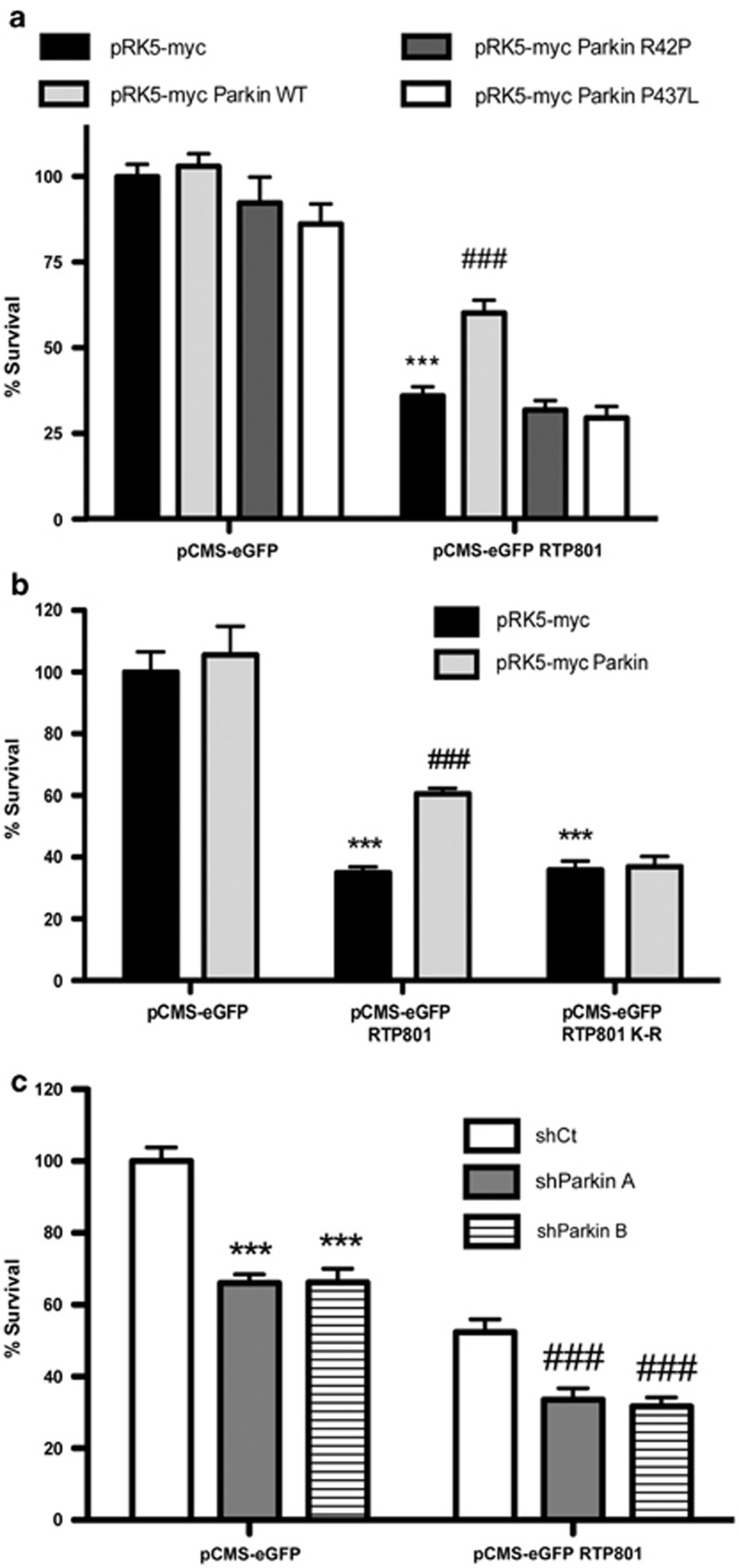
Parkin protects from RTP801-induced cell death. (**a**) Ectopic parkin WT protects from the cell death caused by RTP801. Neuronal PC12 cells were co-transfected with pRK5-myc, pRK5-myc Parkin WT, pRK5-myc Parkin R42P or pRK5-myc Parkin P437L constructs, along with either pCMS-eGFP or pCMS-eGFP RTP801 constructs. Twenty-four hours later, eGFP+ surviving cells were scored under fluorescence microscopy. Values represented as mean ± S.E.M. of at least three independent experiments in triplicate in each condition. ANOVA Bonferroni's multiple comparison test, ***P<0.001 *versus* pRK5-myc/pCMS-eGFP, ^###^*P*<0.001 *versus* pRK5-myc/pCMS-eGFP RTP801. (**b**) Parkin protects neuronal PC12 cells from RTP801-induced cell death by mediating its ubiquitination. In NGF-differentiated PC12 cells, we co-transfected pRK5-myc or pRK5-myc Parkin, along with one of the pCMS-eGFP constructs, either empty or containing RTP801 or RTP801 K-R. Twenty-four hours later, cells were fixed and immunostained, and eGFP+ surviving cells were scored under fluorescence microscopy. Values represented as mean ± S.E.M. of at least three independent experiments in triplicate in each condition. ANOVA Bonferroni's multiple comparison test, ***P<0.001 *versus* pRK5-myc/pCMS-eGFP, ^###^*P*<0.001 *versus* pRK5-myc/pCMS-eGFP RTP801. (**c**) Knocking down parkin exacerbates RTP801-mediated cell death. PC12 cells differentiated with NGF were first transfected with a scrambled negative control shRNA (shCt) or two different parkin shRNAs (shParkin A or B), and after 72 h, cultures were transfected with pCMS-eGFP or pCMS-eGFP RTP801 constructs. Twenty-four hours after the second transfection, ZsGreen+/eGFP+ surviving cells were scored under fluorescence microscopy. Values represented as mean ± S.E.M. of at least three independent experiments in triplicate in each condition. ANOVA with Bonferroni's multiple comparison test, ***P<0.001 *versus* shCt/pCMS-eGFP, ^###^*P*<0.001 *versus* shCt/pCMS-eGFP RTP801. In each panel, values represent mean ± S.E.M. of, at least, three independent experiments with four replicates per condition

Previous studies showed that RTP801 is pro-apoptotic when overexpressed in neuronal PC12 cells^17, 19^ and sympathetic neurons.^20^ This raised the question of whether parkin protects from elevated RTP801 expression. In neuronal PC12 cells, overexpressing wild-type parkin alone did not affect survival, whereas the two parkin mutants, R42P (mutated in the UBL domain) or P437L (mutated in the RING2 domain), with severely compromised E3 ligase activity, showed a slight, but not significant decrease in viability. When RTP801 was overexpressed alone, around 60% of the cells died, as previously reported.^17, 19^ However, ectopic wild-type parkin, but not the mutated parkin forms, counteracted RTP801-induced cell death (Figure 6a).

To confirm that parkin protects from ectopic RTP801 by poly-ubiquitinating it, we generated a non-ubiquitinable form of RTP801 (RTP801 K-R), in which we substituted its six lysines (K) with arginines (R). In neuronal PC12 cells, overexpressed RTP801 K-R accumulated to a significantly higher degree than overexpressed WT RTP801 (Supplementary Figure S6), which is consistent with the role of ubiquitination and proteasomal degradation in regulating RTP801 expression. Both WT and RTP801 K-R induced similar levels of cell death (Figure 6b). However, when these vectors were overexpressed along with parkin, in contrast to its protection from WT RTP801, ectopic parkin was incapable of preventing cell death induced by RTP801 K-R (Figure 6b). These results are consistent with the idea that RTP801 ubiquitination by parkin is necessary for inhibiting RTP801 toxicity.

We next investigated whether parkin knockdown enhances ectopic RTP801 toxicity. In NGF-differentiated PC12 cells, parkin knockdown alone induced around 30–35% cell death (Figure 6c). Ectopic RTP801 alone caused 50% cell loss, whereas, interestingly, parkin knockdown significantly enhanced such cell death to about 70% (Figure 6c).

### Parkin diminishes RTP801 elevation and protects against 6-OHDA

**Figure 7:**
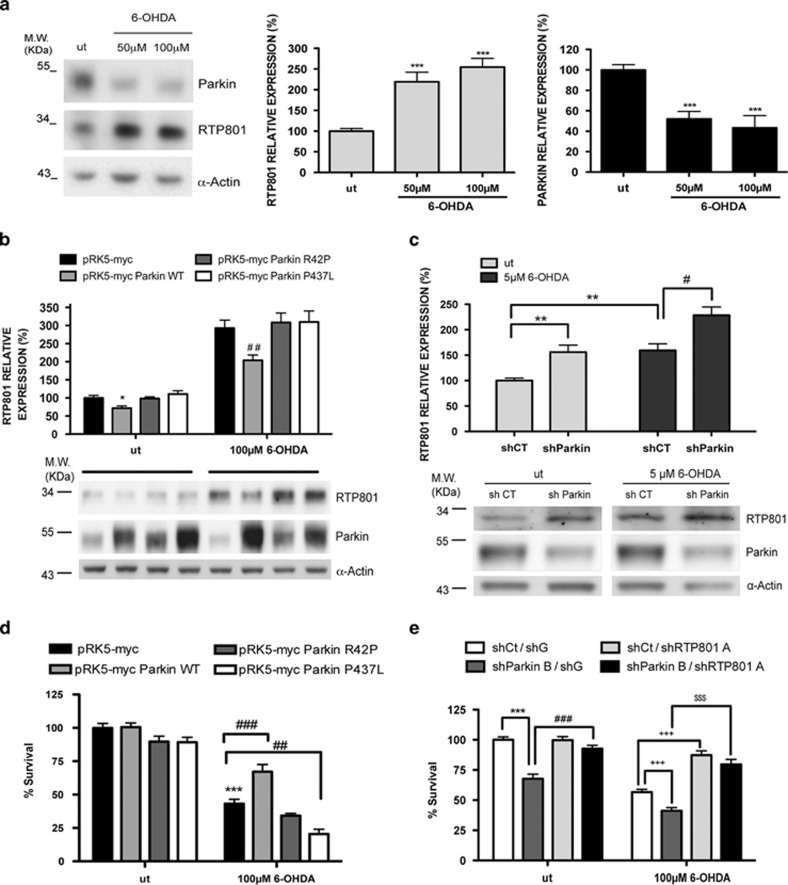
Parkin decreases elevation of RTP801 and protects from 6-OHDA. (**a**) RTP801 and parkin protein levels after 6-OHDA exposure. Neuronal PC12 cells were exposed to 50 or 100 *μ*M 6-OHDA for 16 h. Cell extracts were subjected to western immunoblotting. Membranes were probed with antibodies against parkin and RTP801, and then reprobed for *α*-actin as protein loading control. Right panels represent quantification of parkin and RTP801 densitometric values (mean ± S.E.M.) normalized to *α*-actin, of three independent experiments. ANOVA with Bonferroni's multiple comparison test, ***P<0.001 *versus* untreated control cultures. (**b**) Ectopic parkin decreases 6-OHDA-induced RTP801 protein. Neuronal PC12 cells were transfected with pRK5-myc, pRK5-myc Parkin WT, pRK5-myc Parkin R42P or pRK5-myc Parkin P437L plasmids. Forty-eight hours later, cultures were exposed to 100 *μ*M 6-OHDA for 16 h. Cell extracts were harvested and subjected to western immunoblotting. Membranes were incubated with anti-parkin and anti-RTP801 antibodies. The same membranes were then reprobed with anti-*α*-actin antibody as loading control. Upper panel shows RTP801 densitometries representing values as mean ± S.E.M. of at least three independent experiments. ANOVA with Bonferroni's multiple comparison test, *P<0.05 *versus* untreated pRK5-myc, ^##^*P*<0.01 *versus* 6-OHDA-treated pRK5-myc. (**c**) Parkin knockdown increases RTP801 levels induced by 6-OHDA. Rat sympathetic neurons were infected with lentiviruses containing either a scrambled negative control shRNA (shCT) or a pool of three different shRNA constructs against parkin (shParkin). Six days later, cultures were treated with 5 *μ*M 6-OHDA for 16 h. Cell extracts were harvested and subjected to western immunoblotting. Membranes were probed with antibodies against parkin, RTP801 and *α*-actin as a loading control. Upper panel shows RTP801 quantification (mean ± S.E.M.) normalized to *α*-actin, of three independent experiments. ANOVA with Bonferroni's multiple comparison test, **P<0.01 *versus* untreated shCT, ^#^*P*<0.05 *versus* 6-OHDA-treated shCT. (**d**) Parkin protects from 6-OHDA-induced cell death. Neuronal PC12 cells were co-transfected with pRK5-myc/pCMS-eGFP, pRK5-myc Parkin WT/pCMS-eGFP, pRK5-myc Parkin R42P/pCMS-eGFP or pRK5-myc Parkin P437L/pCMS-eGFP constructs (4:1) and 48 h later cultures were treated with 100 *μ*M 6-OHDA. Twenty-four hours after 6-OHDA exposure, eGFP+ surviving cells were scored under fluorescence microscopy. Values represent mean ± S.E.M. of at least three experiments with six replicates in each condition. ANOVA with Bonferroni's multiple comparison test, ***P<0.001 *versus* untreated pRK5-myc/pCMS-eGFP, ^##^*P*<0.01 and ^###^*P*<0.001 *versus* 6-OHDA-treated pRK5-myc/pCMS-eGFP. (**e**) Parkin knockdown sensitizes to 6-OHDA-induced cell death in an RTP801-dependent manner. Neuronal PC12 cells were transfected with parkin shRNA (shParkin B) or its scrambled negative control (shCt), along with a shRNA sequence against RTP801 (shRTP801) or its corresponding shRNA scrambled control sequence (shG). After 72 h of transfection, cultures were treated with 100 *μ*M 6-OHDA. Twenty-four hours after treatment cells were fixed and ZsGreen+/eGFP+ surviving cells were scored under fluorescence microscopy. Values represent mean ± S.E.M. of at least three experiments with three replicates in each condition. ANOVA with Bonferroni's multiple comparison test, ***P<0.001 *versus* untreated shCt/shG, ^###^*P*<0.001 *versus* untreated shParkin B/shG, ^+++^*P*<0.001 *versus* 6-OHDA-treated shCt/shG and ^$$$^*P*<0.001 *versus* 6-OHDA-treated shParkin B/shG. ut = untreated

Parkin is protective in a variety of neurodegeneration models, including the 6-OHDA model of PD.^13, 14^ Our previous studies indicate that induced RTP801 mediates neurotoxin-promoted death of dopaminergic neurons *in vitro* and *in vivo*.^19, 20, 23^ Therefore, we next determined whether there is a relationship between parkin and RTP801 levels in the neuronal PC12 cell 6-OHDA model. 6-OHDA significantly induced endogenous RTP801 protein^19^ and endogenous parkin levels were diminished by nearly half^33^ (Figure 7a). Moreover, we found that transfecting neuronal PC12 cells with WT parkin, but not the R42P and P437L parkin mutants, significantly decreased elevation of RTP801 protein induced by 6-OHDA (Figure 7b).

To verify our findings with primary neurons, we turned to sympathetic neurons, a population that degenerates in PD. We observed parkin knockdown by about 50% in cultured rat sympathetic neurons with lentivirally expressed shRNAs against parkin. This raised basal RTP801 levels by about the same magnitude (Figure 7c). Moreover, parkin knockdown in these neurons significantly increased RTP801 protein induction by 6-OHDA (Figure 7c). These results support our observations in neuronal PC12 cells that RTP801 knockdown increases RTP801 levels and extend them to show that parkin knockdown exacerbates the RTP801 response to 6-OHDA in neurons (see Figure 2c).

We next examined whether ectopic parkin, which decreases endogenous RTP801 upregulation (Figures 7a and b), is neuroprotective in the 6-OHDA model. In neuronal PC12 cells, overexpressed WT parkin, but not the two parkin mutants, significantly reduced 6-OHDA-induced death (Figure 7d). Note that the parkin P437L mutant decreased cell viability after 6-OHDA exposure, possibly by acting as a dominant-negative.

Finally we explored whether parkin knockdown sensitizes neuronal PC12 cells to 6-OHDA toxicity and whether this depends on RTP801. Parkin knockdown induced cell death in untreated cultures (see also Figure 6c) and enhanced 6-OHDA-promoted toxicity (Figure 7e and Supplementary Figure S7). When we simultaneously knocked down parkin and RTP801, we prevented parkin knockdown-induced cell death and its sensitization to 6-OHDA. These findings indicate that the toxic effects of parkin loss of function require the presence of RTP801.

Taken together, these results indicate that in cellular models of PD, parkin protects, at least in part, by preventing RTP801 elevation.

## Discussion

Here, we identified a new substrate of parkin. We show that parkin mediates RTP801/REDD1 poly-ubiquitination, facilitates its proteasomal degradation and that endogenous parkin and RTP801 interact in living cells. We also show that parkin loss of function elevates RTP801 protein in cellular and animal models. This is supported by observations of elevated RTP801 in both postmortem PD brains and fibroblasts from patients with parkin mutations. Furthermore, overexpression of WT parkin, but not its inactive mutated forms, protects neuronal cells from death when RTP801 is overexpressed or induced with 6-OHDA. We thus propose that one mechanism by which parkin exerts neuronal protection is by preventing accumulation of lethal RTP801 protein levels.

Our data indicate that RTP801 is poly-ubiquitinated and targeted to the ubiquitin proteasome system (UPS). These results agree with others that RTP801 degradation depends on the UPS.^21, 22, 23^ UPS alterations are a common feature in cellular and animal models of PD and in the disease itself.^34, 35^ Based on our results, UPS impairment associated with PD could contribute, in part, to RTP801 elevation in both sporadic PD and AR-JP.

We showed that parkin poly-ubiquitinates RTP801, both *in vitro* and *in vivo*. The only presently described E3 ligase for REDD1/RTP801 is CUL4A-DDB1 in HEK293 and MCF-7 cells.^21^ However, Zhao *et al.*^36^ could not confirm this result. Whether Cul4A-DDB1 is relevant in the context of PD is presently unknown.

Parkin and its ligase activity have been linked to both familial and sporadic PD. Mutations in *PARK2*,^5, 6^ S-nitrosylation^7^ and oxidative^8^ or dopaminergic stress^9^ critically compromise parkin solubility and E3 ligase activity. Parkin enzymatic impairment has been proposed to produce toxic accumulation of its substrate proteins, which in turn causes neurodegeneration.

Many proteins have been proposed as parkin substrates; however only a few have been confirmed. Dawson *et al.*^37^ proposed that a ‘true' parkin substrate should accumulate in both AR-JP and sporadic PD as well as in animal models of parkin inactivation such as PaKO mice and the MPTP toxin model.^37^ Our previous^19, 23^ and present work appears to fulfill these requirements^37^ for RTP801. Additional parkin substrates have been reported^38^ and these, along with RTP801, could represent accumulated proteins that contribute to neuron degeneration associated with parkin loss of expression or function.

We found a relatively modest, but significant, increase of RTP801 in PaKO mice. These animals have motor and non-motor behavioral impairments,^39^ deficient synaptic transmission,^40^ reduced mitochondrial respiration and increased oxidative damage,^41^ and loss of DA neurons after 2 years of age.^42, 43^ The PaKO mice examined in our study were only 8-month old. Our data raise the possibility that early RTP801 accumulation could contribute to the impairments cited above and to neuron death at an older age. Also, in younger PaKO mice, other ligases may compensate for parkin loss and maintain levels of substrate proteins, such as RTP801. The relatively small increase in RTP801 seen in 8-month-old PaKO mice is also consistent with, and could potentially account for, the lack of neuron death at this age.

In postmortem sections from two PD cases with *PARK2* gene mutations, NM+ neurons showed higher levels of RTP801 expression. One case involved compound heterozygous mutations and behaved as a homozygous parkin mutant, with early onset of disease. However, the second case had a simple heterozygous mutation in the parkin gene, with late disease onset. Both cases showed elevated RTP801 levels as in sporadic cases. This could reflect findings that loss of only one copy of the parkin gene can be a risk factor for PD.^10, 44^ To extend our results, fibroblasts from six AR-JP patients with *PARK2* gene mutations were assessed. Cultured skin fibroblasts are very useful to study genetic PD mechanisms.^45^ We reasoned that mutations in the *PARK2* gene should influence RTP801 turnover in cells in addition to neurons. We observed that RTP801 protein levels approximately doubled in AR-JP patient fibroblasts compared with controls, and that the RTP801 response to etoposide or epoxomycin was exaggerated in AR-JP fibroblasts. This is consistent with the idea that parkin loss of function elevates RTP801 in both neuronal and non-neuronal cells. However, as fibroblasts are proliferative, RTP801 does not induce their cell death as it does in non-dividing neurons.^17, 19^

Our previous work demonstrated that RTP801 was upregulated in cellular and animal models of PD and in NM+ nigral neurons of sporadic PD patients. It also showed that RTP801 elevation is sufficient to mediate neuron cell death, and that RTP801 is required for neuron death in several PD models.^19^ Hence, as parkin loss of function leads to RTP801 elevation, it is logical to suggest that this effect contributes to neuronal degeneration and cell death in the context of PD. RTP801 protein elevation can be the end point of two different processes; one being gene activation by PD-associated neuronal stress,^18, 19^ and the other, defective RTP801 degradation due to impaired parkin activity. RTP801 may therefore contribute, with other parkin substrates, to neuron death and degeneration in both sporadic and parkin-associated PD.

Parkin protects from neuron toxicity associated with multiple stressors including mutant alpha synuclein,^46^ kainate acid excitotoxicity^12^ as well as dopamine^47^ and 6-OHDA.^14^ In consonance with this, we observed that WT parkin, but not its mutated forms, decreased RTP801-induced cell death of neuronal PC12 cells. Significantly, the neuroprotective effect of parkin was lost for non-ubiquitinable form of RTP801. Ectopic parkin protected against 6-OHDA, and this protection was accompanied by a decrease in RTP801 protein. Moreover, parkin knockdown diminished cell survival, and sensitized cells against 6-OHDA. Interestingly, RTP801 knockdown abrogated this sensitization and restored cell survival. These results strongly suggest that parkin is protective, at least in part, by mediating RTP801 proteasomal degradation. Neuroprotective effects of parkin appear due, at least in part, to its effects on mitochondria, by regulating cytochrome c release and apoptosis.^48^ A portion of RTP801 protein is also translocated to mitochondria.^49^ This suggests that clearance of RTP801 by parkin may have direct effects on mitochondrial function as well as on mTOR/Akt signaling.

Abrogation of RTP801 expression with shRNAs or pharmacologic agents has been demonstrated to be a useful strategy for neuroprotection. In cellular models of PD, RTP801 shRNAs protected both sympathetic neurons and neuronal PC12 cells from neurotoxins.^19^ RTP801 knockdown also has been successful in treating ocular diseases like macular degeneration.^50^ Pharmacologically, rapamycin, by partially inhibiting mTOR, interferes with RTP801 translation and is protective in cellular and animal models of PD.^23^ Our data indicate that parkin contributes to decreasing cellular RTP801 protein levels. Therefore, means to elevate parkin levels or activity in neurons would be anticipated to provide neuroprotection, at least, in part, via degradation of RTP801.

In conclusion, our work in cellular and animal models and in human samples strongly indicates that RTP801 is a substrate of parkin and that RTP801 elevation due to parkin loss of function in both AR-JP and sporadic PD may contribute to neurodegeneration. Any genetic conditions or stress situations that compromise parkin activity have the potential to produce a toxic accumulation of RTP801. This is relevant to design of therapeutic approaches to promote neuron survival and block neurodegeneration in both sporadic and AR-JP.

## Materials and methods

### Antibodies, plasmids and materials

Rabbit polyclonal anti-RTP801 antibody (used for WB and immunohistochemistry) was purchased from Proteintech Group (Chicago, IL, USA). Mouse monoclonal anti-RTP801 (used for immunoprecipitation) was purchased from Bethyl Laboratories (Montgomery, TX, USA). Monoclonal mouse antibody anti-parkin used for WB and immunohistochemistry was purchased from Abcam (Cambridge, UK). Monoclonal mouse anti-parkin antibody (used for immunoprecipitation), polyclonal rabbit anti-myc (used for WB), monoclonal mouse anti-myc (used for immunoprecipitation) and anti-HA tag were obtained from Cell Signaling Technology (Danvers, MA, USA). Mouse monoclonal antibody against GFP and rabbit polyclonal antibodies against parkin and ERK1 were obtained from Santa Cruz Biotechnology (Dallas, TX, USA). Anti-*α*-actin antibody was purchased from MP Biomedicals (Santa Ana, CA, USA). Goat anti-mouse or anti-rabbit secondary antibodies conjugated to horseradish peroxidase were obtained from Pierce Thermo Fisher Scientific (Rockford, IL, USA). Goat anti-mouse or anti-rabbit secondary antibodies conjugated with Alexa 488 or Alexa 568 were purchased from Life Technologies (Grand Island, NY, USA). Peroxidase-conjugated goat anti-IgG light chain-specific secondary antibody was obtained from Jackson ImmunoResearch Laboratories (West Grove, PA, USA).

The pCMS-eGFP RTP801 construct was generated as previously described.^19^ The pcDNA3 HA-ubiquitin construct was purchased from Addgene (Cambridge, MA, USA). The constructs pEGFP-C2, pEGFP-C2-parkin, pRK5-myc, pRK5-myc Parkin and the parkin point mutation pRK5-myc Parkin P437L were a kind gift from Dr. Serge Przedborski (Columbia University, New York, NY, USA). The parkin point mutant pRK5-myc Parkin R42P and the parkin deletion pRK5-myc Parkin ΔR2 were kindly provided by Dr. Leonidas Stefanis (Academy of Athens, Biomedical Research Foundation, Athens, Greece). All constructs were verified by DNA sequencing. Epoxomycin, MG132 and etoposide were purchased from Calbiochem Merck Millipore (Billerica, MA, USA), Chloroquine and CHAPS were obtained from Sigma-Aldrich (St. Louis, MO, USA) and 6-OHDA was purchased from Tocris Bioscience (Bristol, UK).

### Directed mutagenesis

The pCMS-eGFP RTP801 K-R construct was obtained from the pCMS-eGFP RTP801 construct by mutating all the lysines (K) to arginines (R) using the QuickChange Lightning II multi site-directed mutagenesis (Agilent Technologies, Santa Clara, CA, USA), with the following primers: K126R, 5′-GAGCCAGGTGGGCAGGGAACTCCTGC-3′ K152R, 5′-GTGTGGAGCAAGGCAGGAGCTGCCATAGTGT-3′ K185R, 5′-GCCTCTGGCCCAGGATCCAGGGCCT-3′ K215R-K216R-K217R, 5′-CACCGGCTTCAGAGTCATCAGAAGGAGACTCTACAGCTCCGAG-3′ All new constructs were verified by DNA sequencing.

### shRNA production

shG-scrambled control RNA and shRTP801 were generated as previously described.^51^ Two different parkin shRNAs were prepared in a ZsGreen expressing pSIREN vector using the Knockout RNAi Systems (Clontech Laboratories, Mountain View, CA, USA) according to the manufacturer's instructions, based on the following sequences: shParkin A, 5′-AACAACAGAGTATCGTTCACA-3′ shParkin B, 5′-ATCGTTCACATAGTACAGAGA-3′ shParkin 1, 5′-AGCTCCATCACTTCAGGATCC-3′ (as described in Sun *et al.*^33^); shParkin 2, 5′-ATCACCTGACAGTACAGAACT-3′. The scrambled negative control shRNA (shCt) was provided by the same kit with the sequence: 5′-GTGCGTTGCTAGTACCAAC-3′. shParkins A, B and 2 were specific for rattus norvegicus parkin, whereas shParkin 1 was specific for rat and mouse.

### Lentivirus preparation

Viral particles for neuronal PC12 infection were prepared as previously described.^33^

### Cell culture

PC12 cells were cultured and treated with NGF as described previously.^52^ For NGF treatment, the cells were cultured in RPMI 1640 medium (Gibco Life Technologies, Grand Island, NY, USA) supplemented with 1% heat-inactivated horse serum (Sigma-Aldrich), penicillin/streptomycin, and 50 ng/ml recombinant human *β*-NGF (Alomone Labs, Jerusalem, Israel; or kindly provided by Genentech, South San Francisco, CA, USA) for 7–8 days, in a 7.5% CO_2_ atmosphere at 37 °C.

Neonatal rat superior cervical ganglion sympathetic neurons were cultured as previously described.^19, 53^

HEK293 cells were maintained in DMEM medium (Gibco Life Technologies) supplemented with 10% fetal calf serum (Gibco Life Technologies) and penicillin/streptomycin (Gibco Life Technologies) in a 5% CO_2_ atmosphere at 37 °C.

Human fibroblasts derived from six diagnosed AR-JP patients with *PARK2* dysfunctional mutations and six control individuals were cultured in DMEM medium supplemented with 10% fetal calf serum and penicillin/streptomycin in a 5% CO_2_ atmosphere at 37 °C. These fibroblasts were obtained from the Neurology Service-Movement Disorders Biorepository (IDIBAPS, Hospital Clinic, Universitat de Barcelona, Barcelona, Catalonia, Spain). Fibroblasts were used between 5 and 6 passages *in vitro*.

### Transfection, treatments and viral infection

Neuronal PC12 and HEK293 cells were transfected with Lipofectamine 2000 (Invitrogen Life Technologies, Grand Island, NY, USA) according to the manufacturer's instructions.

6-OHDA treatments were performed after 6–7 days of culture in NGF-differentiated PC12, and at day *in vitro* (DIV) 9 in primary rat sympathetic neuronal cultures. Medium was changed right before treatments.

Neuronal PC12 cells were infected at DIV3 and at a multiplicity of infection (MOI) of 3. Primary neonatal rat SCG sympathetic neurons were infected at DIV 3 and at a MOI of 2, with lentiviral particles containing a pool of three different shRNAs constructs against rat parkin (shParkin) or a scrambled control sequence (shCT), both obtained from Santa Cruz Biotechnologies. After 18–20 h of infection, medium containing remaining lentiviral particles was removed and replaced by fresh medium.

### Quantitative reverse transcription-PCR

Each sample of total RNA was isolated from neuronal PC12 cells by using the High Pure RNA Isolation Kit (Roche Diagnostics Corporation, Indianapolis, IN, USA). cDNA was transcribed from total RNA with the Transcriptor First Strand cDNA Synthesis Kit Roche Diagnostics Corporation. Specific primers used for quantitative PCR amplification were as follows: RTP801 forward primer, 5′-GCTCTGGACCCCAGTCTAGT-3′ RTP801 reverse primer, 5′-GGGACAGTCCTTCAGTCCTT-3′ *α*-actin forward primer, 5′-GGGTATGGGTCAGAAGGACT-3′ *α*-actin reverse primer, 5′-GAGGCATACAGGGACAACAC-3′. Quantitative PCR was performed with a 7500 Real Time PCR System (Applied Biosystems, Foster City, CA, USA) using equal amounts of cDNA template for quantitative PCR analysis of RTP801, normalized by *α*-actin. The genes analyzed in this study were examined by the relative expression by means of ΔΔCT.

### WB analysis

Whole-cell extracts or 8-month-old PaKO mouse total brain lysates were collected, prepared and analyzed, as previously described.^19, 28^ Chemoluminiscent images were acquired with a LAS-3000 Imager (Fujifilm, Valhalla, NY, USA).

### Immunoprecipitation

In the experiments where we immunoprecipitated ectopic RTP801 and parkin, cell extracts were collected with cell lysis buffer (Cell Signaling Technology) and pre-cleared with protein A/G sepharose beads (Immunoprecipitation Starter Pack; GE Healthcare Bio-Science, Pittsburgh, PA, USA) for 1 hour at 4 °C. Pre-cleared cell lysates were incubated overnight at 4 °C in rotation, with the corresponding antibody or a normal immunoglobulin (Santa Cruz Biotechnology) as a negative control. Immunocomplexes were incubated with BSA-blocked protein A/G sepharose beads for 4 h at 4 °C in rotation. Then the beads were centrifuged and washed five times with CHAPS buffer (Tris 50 mM; pH 7.4, NaCl 150 mM, MgCl_2_ 10 mM, CHAPS 0.4%), and the immunocomplexes were collected and subjected to WB. The cross-linking agent DSP (Gibco Life Technologies) was used according to the manufacturer's instructions. In order to immunoprecipitate endogenous RTP801 and parkin, cell extracts were collected with cell lysis buffer and incubated overnight at 4°C in rotation, with the corresponding antibody or a control antibody (GFP) as a negative control. Immunocomplexes were incubated with protein A (parkin) or protein G (RTP801) for 2 h at 4 °C in rotation. Then the beads were centrifuged and washed four times with cell lysis buffer, and the immunocomplexes were resolved in a western blot.

### *In vitro* ubiquitination

The assay was performed by using the Ubiquitinylation Kit according to the manufacturer's instructions (Enzo Life Sciences, Farmingdale, NY, USA) with slight modifications. Briefly, (20 mM Tris-HCl; pH 7.5, 1 mM dithiothreitol, 20 U/ml inorganic pyrophosphatase, 5 mM Mg-ATP, 0.1 *μ*M His_6_-tagged recombinant human ubiquitin-activating enzyme E1, 2.5 *μ*M biotinylated ubiquitin), His_6_-tagged recombinant human ubiquitin-conjugating enzyme UbcH7 E2 (Enzo Life Biosciences), His_6_-tagged recombinant human parkin full-lenght (Merck Millipore), N-terminal GST-tagged recombinant human RTP801 full-length (Novus Biologicals, Cambridge, UK), and GST-tagged recombinant human truncated and inactive parkin (1-387) (Novus Biologicals), were added to the reaction buffer, as referred in each condition, and then incubated at 37 °C for 90 min. RTP801 was immunoprecipitated as described above, and equal volumes of each sample were subjected to WB. The blotted membranes were probed for biotinylated ubiquitin using Avidin/Biotin-Horseradish Peroxidase (Ultra-sensitive ABC staining kit; Thermo Fisher Scientific, Waltham, MA, USA) or by anti-RTP801 antibody (Proteintech Group).

### Immunofluorescence

Neuronal PC12 cells were fixed with 4% paraformaldehyde, stained and observed under fluorescence microscopy, as previously described.^20^ Fluorescence from stained cells was quantified with ImageJ software (NIH). Fluorescence for parkin staining (in red) or for RTP801 staining (in red) in each single transfected cell (ZsGreen+) with shParkin A and B was quantified and normalized with the fluorescence signal from non-transfected cells, and percentages were compared with the ones obtained with shControl (shCt). Cells were counter-stained with Hoescht 33342 (Invitrogen Life Technologies) to visualize the nucleus. In survival assays, transfected viable cells were scored by strip counting, as previously described.^19^

### Immunohistochemistry of human sections

Paraffin-embedded sections of postmortem human SNpc and cerebellum from control individuals, parkin heterozygous mutant PD patients, and sporadic PD patients, were obtained from the Neurological Tissue Bank (Biobank-HC-IDIBAPS) and stained as previously described.^19^

### Statistics

All experiments were performed at least in triplicate, and results are reported as means±S.E.M. Student's *t*-test was performed as appropriate and indicated in the figure legends, mostly as unpaired, two-tailed sets of arrays and presented as probability *P*-values, or otherwise stated in the figure legends. One-way or two-way ANOVA with Bonferroni's multiple comparison test were performed to compare multiple groups.

## Abbreviations

6-OHDA: 6-hydroxydopamine
AR-JP: autosomal recessive juvenile parkinsonism
DSP: dithiobis succinimidyl propionate
EGFP: enhanced green fluorescent protein
HMW: high molecular weight
IP: immunoprecipitation
mTOR: mechanistic target of rapamycin
NGF: nerve growth factor
NM+: neuromelanin positive
PaKO: parkin knockout
PKm: parkin mutant
PD: Parkinson's disease
shRNA: short hairpin RNA
SPD: sporadic Parkinson's disease
SNpc: substantia nigra pars compacta
UPS: ubiquitin proteasome system
WB: western immunoblotting
WT: wild type
