# Supplementary figures and images for: Parkin loss of function contributes to RTP801 elevation and neurodegeneration in Parkinson's disease
Source: Cell Death Dis. 2014 Aug 7;5(8):e1364–. doi: 10.1038/cddis.2014.333 (PMC4454308; doi:10.1038/cddis.2014.333)

# S1

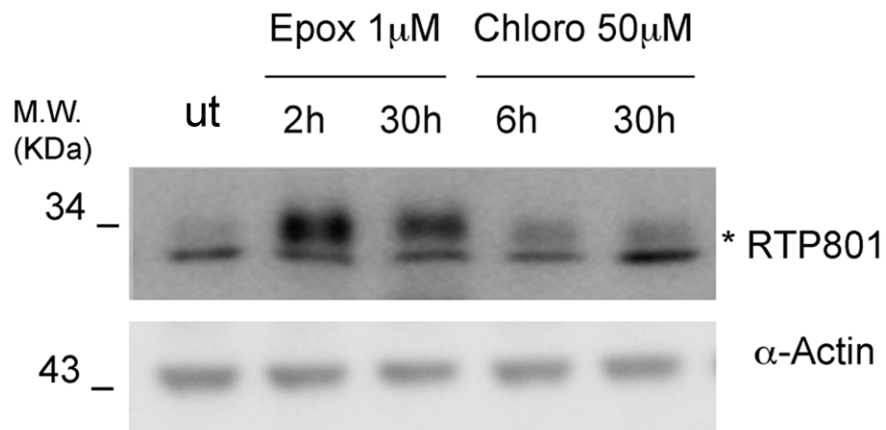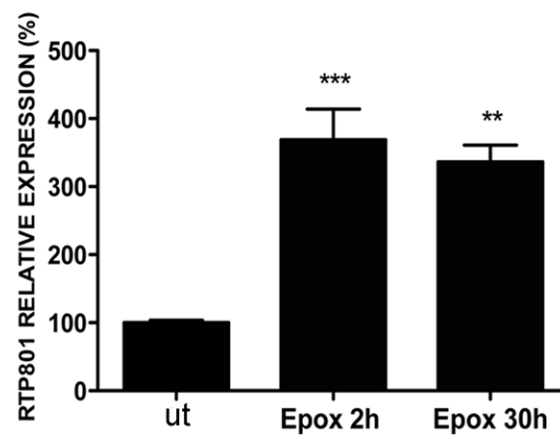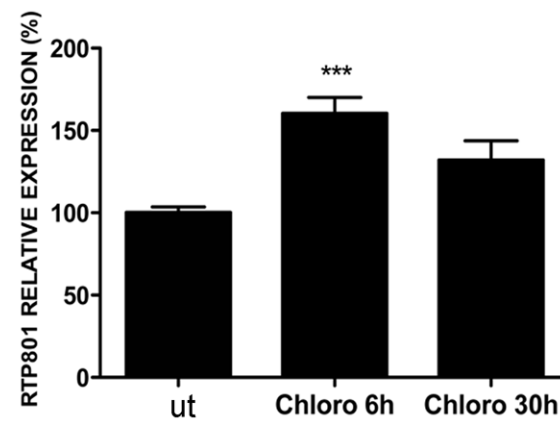

Supplement: Supplementary Figure S1 [file cddis2014333x1.pdf]

S2

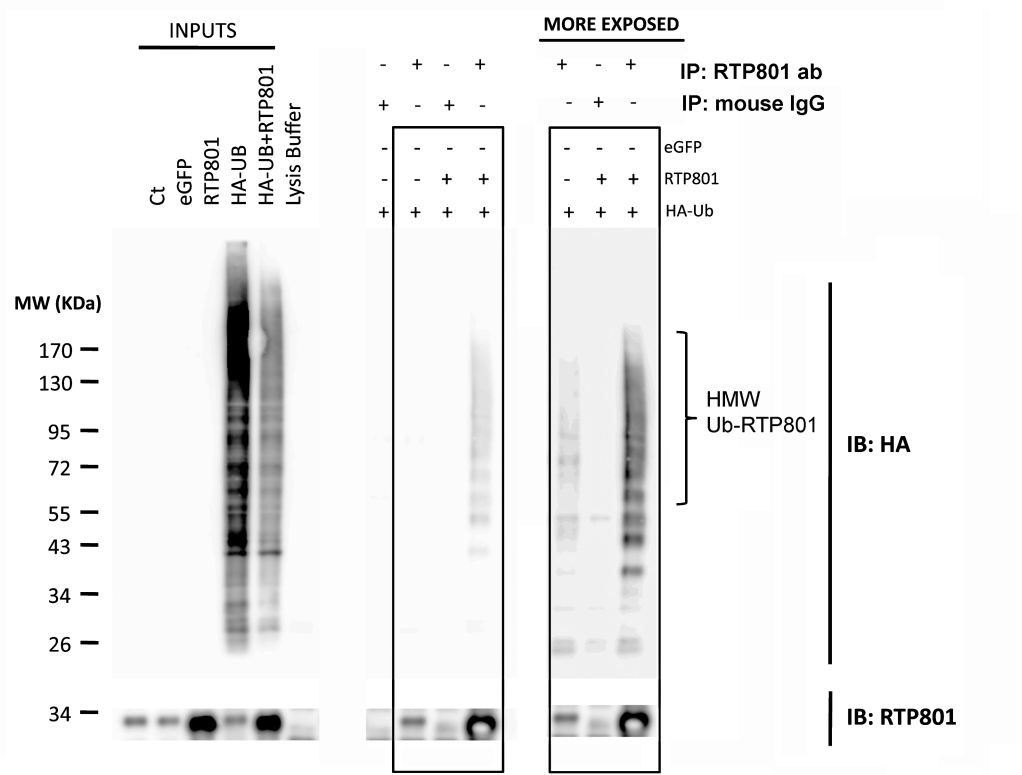

Supplement: Supplementary Figure S2 [file cddis2014333x2.pdf]

S3

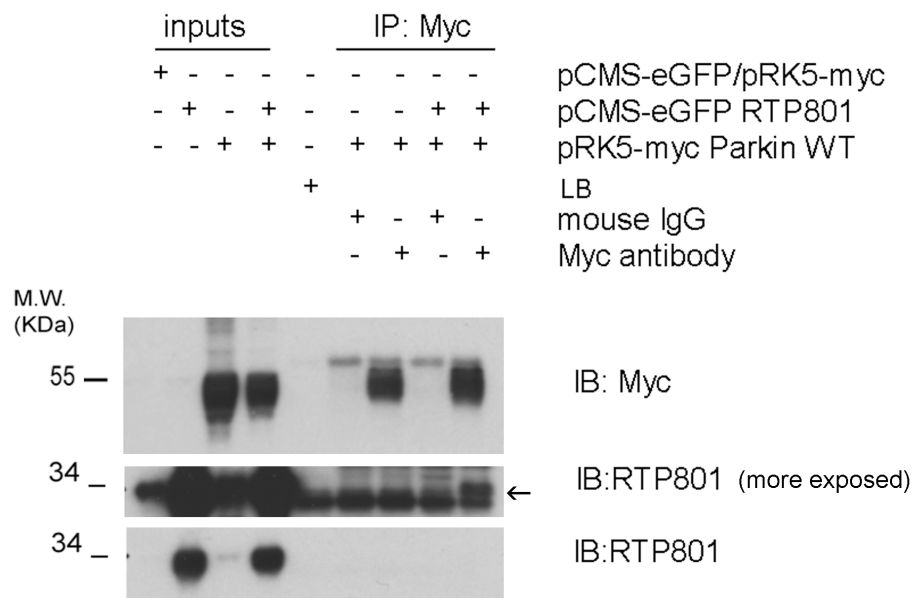

Supplement: Supplementary Figure S3 [file cddis2014333x3.pdf]

S4

a

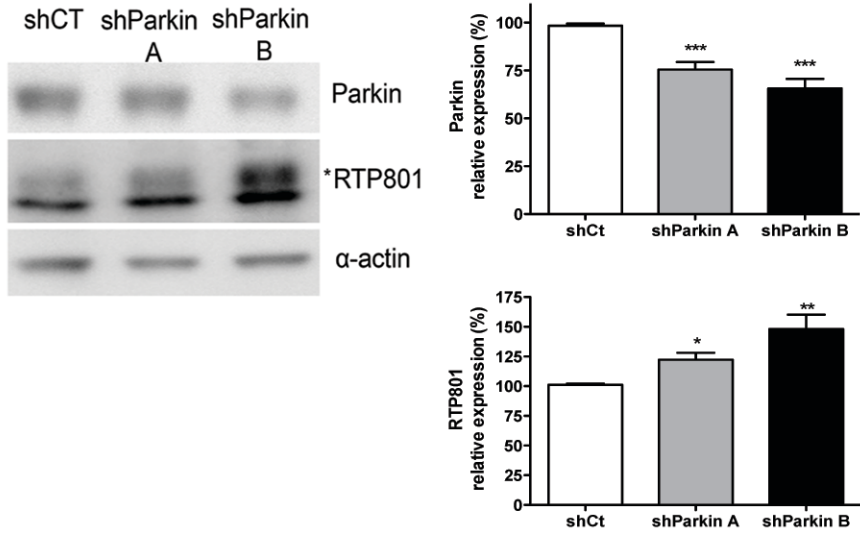

b

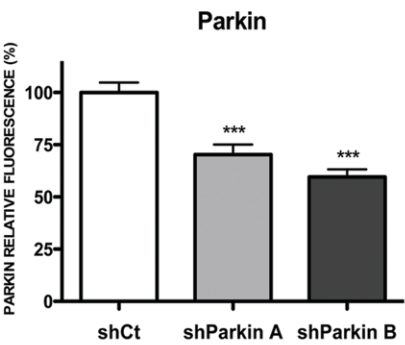

c

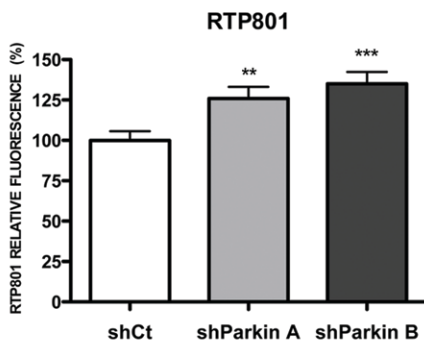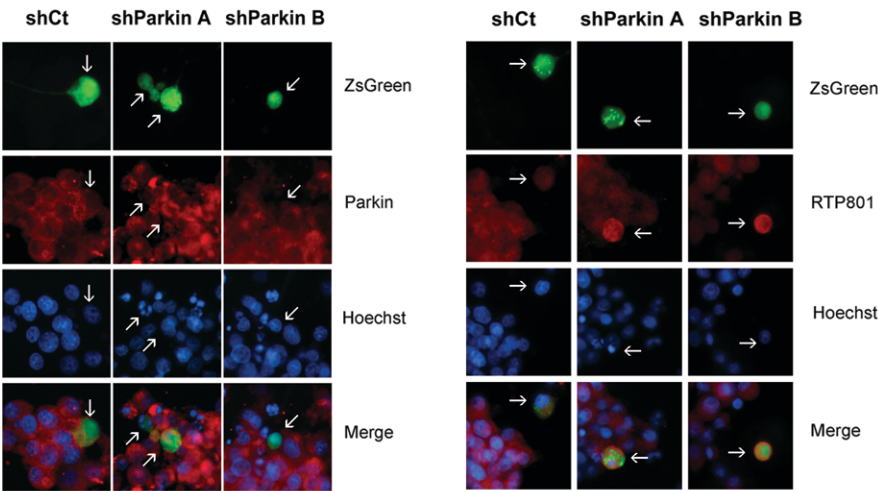

Supplement: Supplementary Figure S4 [file cddis2014333x4.pdf]

S5

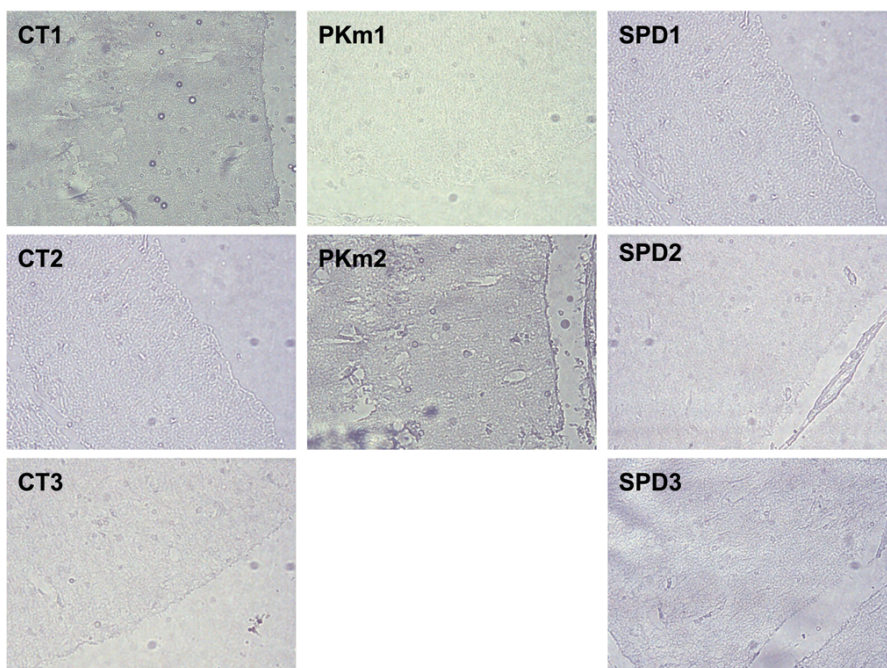

Supplement: Supplementary Figure S5 [file cddis2014333x5.pdf]

S6

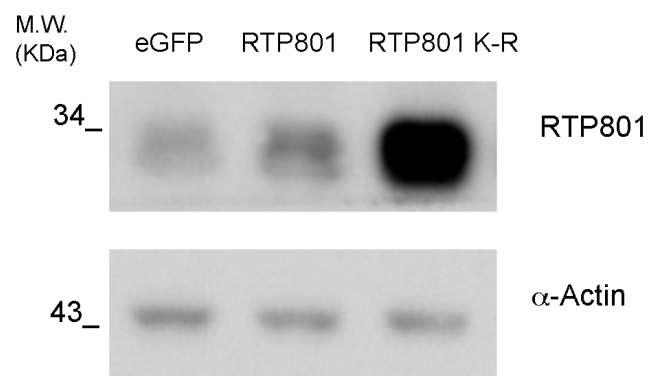

Supplement: Supplementary Figure S6 [file cddis2014333x6.pdf]

S7

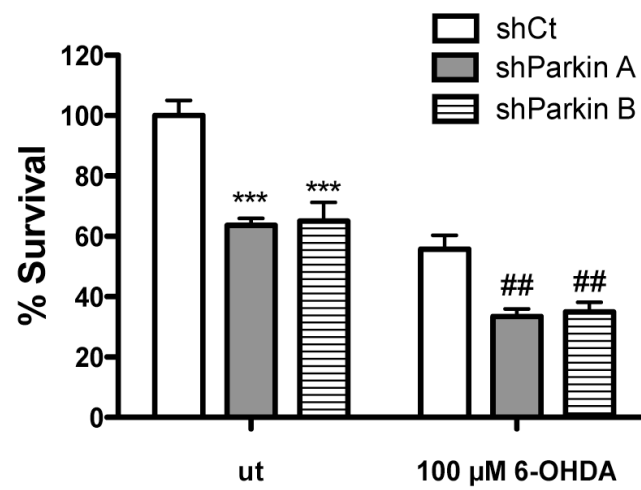

Supplement: Supplementary Figure S7 [file cddis2014333x7.pdf]
