# Supplementary material for: Parkin loss of function contributes to RTP801 elevation and neurodegeneration in Parkinson's disease
Source: Cell Death Dis. 2014 Aug 7;5(8):e1364–. doi: 10.1038/cddis.2014.333 (PMC4454308; doi:10.1038/cddis.2014.333)
Supplement: Supplementary Figure Legends [file cddis2014333x8.doc]

**Figure S1. RTP801 accumulates in neuronal PC12 cells in response to epoxomycin and chloroquine**. Neuronal PC12 cells were exposed to 1 µM epoxomycin for 2 or 30 hours, or 50 µM chloroquine for 6 or 30 hours. Cell lysates were analyzed by Western immunoblotting for RTP801 and α-actin as a loading control. Values represented as mean ± SEM of at least three independent experiments. ANOVA with Bonferroni’s multiple comparison test, ***p*<0.01 and ****p*<0.001 vs untreated cells. ut = untreated.

**Figure S2. RTP801 is poly-ubiquitinated prior to proteasomal degradation.** HEK293 cells were transfected with pCMS-eGFP or pCMS-eGFP RTP801, along with vector expressing HA-tagged ubiquitin (HA-Ub). Twenty-four hours later, cultures were exposed to epoxomycin 2 hours prior to harvesting. RTP801 was immunoprecipitated and immuno-complexes along with whole cell lysates as inputs, were analyzed by Western immunoblot for HA-tag and RTP801. Note the high molecular weight (HMW) smears that appear from 72 KDa to 170KDa, in both ectopic and endogenous (more exposed right panel) RTP801 lanes. Membranes were reprobed with anti-RTP801 antibody to confirm that RTP801 was immunoprecipitated, in comparison to non-specific mouse immunoglobulins (Mouse Igg).

**Figure S3. RTP801 co-immunoprecipitates with parkin.** HEK293 cells were co-transfected with pRK5-myc Parkin and pCMS-eGFP RTP801. After myc immunoprecipitation, the samples were analyzed by Western immunoblotting for RTP801 to detect the interaction, and for myc as an IP control. The RTP801 band is pointed out by an arrow, in the more-exposed panel. A representative Western blot is shown from a pool of at least three independent experiments. LB = Lysis Buffer.

**Figure S4. Knocking down parkin elevates RTP801.** Naïve (a) and NGF-differentiated (b & c) PC12 cells were transfected with a scrambled negative control shRNA (shCt) or two different parkin shRNAs (shParkin A or B), and after five days, cultures were assessed by Western immunoblotting (a) or immunostaining (b & c) for parkin and RTP801. For Western blot quantification, membranes were reprobed with anti-α-actin antibody, as a protein loading control. Upper right panel shows densitometries representing values as mean ± SEM of at least three independent experiments. Specific bands are pointed out by an asterisk. Student’s t-test with Welch’s correction, **p*<0.05, ***p*<0.01 and ****p*<0.001 vs shCt. The relative immunofluorescence signal for parkin and RTP801 in transfected cells (ZsGreen+) was quantified, and values represented as mean ± SEM of at least three independent experiments. Transfected cells are indicated by white arrows. Student’s t-test, ***p*<0.01 and ****p*<0.001 vs shCt.

**Figure S5. RTP801 levels in cerebellum of parkin mutant and idiopathic PD patients.** Paraffin-embedded cerebellar sections from control individuals (CT1, CT2 and CT3), parkin simple heterozygous mutant patient (PKm1), parkin compound heterozygous mutant (PKm2) and sporadic PD patients (SPD1, SPD2 and SPD3) were immunostained for RTP801 (grey-blue) under the same conditions.

**Figure S6. RTP801 K-R mutant accumulates in neuronal PC12 cells.** Neuronal PC12 cells were transfected with pCMS-eGFP, pCMS-eGFP RTP801 or pCMS-eGFP RTP801 K-R constructs. Twenty-four hours later, cell extracts were harvested and subjected to Western immunoblotting. Membranes were incubated with anti-RTP801 antibodies and then reprobed with anti-α-actin antibody, as protein loading control. A representative Western blot is shown from a pool of at least three independent experiments.

**Figure S7. Loss of parkin expression sensitizes neuronal PC12 cells to 6-OHDA-induced cell death.** Neuronal PC12 cells were transfected with a scrambled negative control shRNA (shCt) or two different parkin shRNAs (shParkin A or B), and after 72 hours, cultures were treated with 100 µM 6-OHDA. Twenty-four hours after treatment, cells were fixed and ZsGreen+ surviving cells were scored under fluorescence microscopy. Values represent mean ± SEM of at least three experiments with four replicates in each condition. ANOVA with Bonferroni’s multiple comparison test, ****p*<0.001 vs untreated shCt, ^##^*p*<0.01 vs 6-OHDA-treated shCt. ut = untreated.
