# Supplementary material for: Parkin loss of function contributes to RTP801 elevation and neurodegeneration in Parkinson's disease
Source: Cell Death Dis. 2014 Aug 7;5(8):e1364–. doi: 10.1038/cddis.2014.333 (PMC4454308; doi:10.1038/cddis.2014.333)
Supplement: Supplementary Table 1 [file cddis2014333x9.pdf]

ID	DISEASE STATUS	MUTATION	GENDER	AGE AT SKIN PUNCH BIOPSY	AGE ONSET OF THE DISEASE
1	CONTROL	none	male	66	-
2	CONTROL	none	female	48	-
3	CONTROL	none	female	47	-
4	CONTROL	none	male	52	-
5	CONTROL	none	female	63	-
6	CONTROL	none	male	42	-
7	AR-JP	Homozygous PARK2 ex3del*	male	49	25
8	AR-JP	Homozygous PARK2 ex2-4del*	male	69	35
9	AR-JP	Compound homozygous PARK2 205-206del2/ex6del*	female	44	32
10	AR-JP	Homozygous PARK2 ex5-6del*	female	63	27
11	AR-JP	Compound homozygous CRG ex1del/PARK2 ex6del*	male	35	9
12	AR-JP	Compound homozygous PARK2 ex2dup/ex6del*	male	49	15

Table 1. Human fibroblasts donor information.
(*breakpoint not mapped)
