# Supplementary material for: Parkin loss of function contributes to RTP801 elevation and neurodegeneration in Parkinson's disease
Source: Cell Death Dis. 2014 Aug 7;5(8):e1364–. doi: 10.1038/cddis.2014.333 (PMC4454308; doi:10.1038/cddis.2014.333)
Supplement: Supplementary Table 2 [file cddis2014333x10.pdf]

PATIENT ID	MUTATION	GENDER	AGE	TIME POST MORTEM (H)
CT 1	-	female	74	3:40
CT2	-	female	81	23:30
CT3	-	male	64	10:00
SPD1	-	male	81	5:00
SPD2	-	male	88	15:00
SPD3	-	male	77	12:00
PKM1	simple heterozygous C255deletionA	female	71	5:30
PKM2	compound heterozygous exon 3-6 deletion 472 inst	female	75	6:30

Table 2. Human brain samples information

CT1, CT2 and CT3: control individuals
SPD1, SPD2 and SPD3: sporadic PD patients
PKM1 and PKM2: Parkin heterozygous mutant PD patients
